# Aerobic Versus Resistance Exercise for Overweight: Is there a Difference in Reporting Quality?

**DOI:** 10.1055/a-2596-2049

**Published:** 2025-07-28

**Authors:** Jonas Rohwer, Burkhard Weisser, Manfred Wegner, Claudia Bünzen

**Affiliations:** 1 Institute of Sport Science, Kiel University, Kiel, Germany

**Keywords:** TIDieR, CERT, exercise interventions, reporting quality, overweight

## Abstract

In the management of overweight, the implementation of exercise helps to create a caloric deficit and to lose weight. Several studies have shown poor reporting quality of exercise interventions for other diseases. Thus, the purpose of this study was to assess the completeness of exercise intervention reporting in randomized controlled trials (RCTs) for the treatment of overweight and to evaluate potential differences between exercise modalities. Two independent reviewers applied two intervention reporting guidelines to 47 RCTs on the management of overweight. The completeness of intervention reporting was evaluated using descriptive statistics. Potential differences in reporting quality between studies using aerobic exercise (AE) vs. studies using combined aerobic and resistance exercise (ARE) were calculated with a χ ^2^ test. Overall, studies completed 61% and 47%, respectively, of the guideline items. The χ ^2^ analysis of exercise modalities showed a significant difference for two items regarding exercise progression (91% AE vs. 38% ARE, p<0.001) and detailed description of exercises (0% AE vs. 50% ARE, p<0.001). Reporting of exercise interventions in the treatment of overweight was found insufficient. The detected differences between exercise modalities imply the need for improved guidelines.

## Introduction

Nearly half of all people worldwide are overweight or obese 1 2 . A major factor causing this is the constant availability of food in most regions of the world in combination with poor diet and lack of physical activity 3 .

Overweight and obesity lead to various health issues like type 2 diabetes, hypertension, coronary heart disease, cancer, asthma, and osteoarthritis 4 5 . Accordingly, mortality rates rise with increasing body mass index (BMI) 6 7 . In addition, overweight and obesity can also be a reason for psychosocial consequences such as stigmatization and discrimination 7 .

The need for treatment is evident. Therefore, experts recommend a lifestyle intervention as a combination of exercise, diet, and behavioral therapy. The exercise intervention should include moderate aerobic exercise of at least 150 min per week on three to five days per week 7 8 9 10 . Furthermore, an increase in non-exercise physical activity is recommended 10 . Apparently, resistance exercise is not very effective for weight loss but for maintenance and gain of lean body mass 9 10 . Different meta-analyses demonstrate a loss of body weight between 1 kg and 3.6 kg through exercise interventions for people with overweight and obesity. Effectiveness increases with longer duration and an additional dietary intervention 11 12 13 14 .

Meta-analyses are the basis for many guideline recommendations as they provide the highest level of scientific evidence 15 . Thus, the primary studies used in meta-analyses should be of high quality to ensure the best possible evidence. Detailed descriptions of exercise modalities and intervention conditions need to be included.

However, several studies showed the reporting quality of exercise interventions as a treatment for different diseases to be insufficient 16 17 18 19 20 21 22 23 24 25 26 . Regarding overweight and obesity, one study exists 27 that found overall reporting quality inadequate using the CONSORT statement 28 . In that study, intervention description was represented by only one item that was described sufficiently in 98% of the studies. Given that one item hardly represents general intervention reporting quality, further research with a more exercise-specific checklist is necessary.

To help with detailed reporting specifically on interventions, the checklists Template for Intervention Description and Replication (TIDieR) 29 and Consensus on Exercise Reporting Template (CERT) 30 were developed. In the present study, we used these checklists to assess the reporting quality of exercise intervention studies for overweight and obesity. Using more specific checklists will provide more meaningful results as only sufficiently reported studies can provide a base for general recommendations on the treatment of obesity.

Additionally, different exercise modalities such as aerobic or resistance exercise require different information to be reported in regard to the motion sequence. While cyclical movements like running or cycling need no further explanation, resistance exercises are often more complex and meant to do in a very specific way to target specific muscle groups. They require a more detailed description for adequate performance. For example, CERT item 8 (detailed description of each exercise to enable replication) demands information such as starting position and range of movement in order to avoid ambiguity or misinterpretation 30 . Potentially, this difference in information being required might lead to a difference in reporting quality.

Meneses-Echavez et al. 22 did not detect major differences in intervention reporting quality between different exercise modalities in exercise trials for cancer. For overweight interventions, there is no scientific evidence.

Thus, the purpose of this study is to determine the reporting quality of exercise intervention studies for the treatment of overweight and, moreover, to examine potential differences in reporting quality between aerobic and resistance exercise studies.

## Methods

### Study selection

To be included in this analysis, studies had to fulfill the criteria of being used in meta-analyses that are cited in current clinical guidelines on the management of overweight. Furthermore, they had to be randomized controlled trials (RCTs) using exercise interventions to reduce body weight and/or BMI of people with overweight. Overweight was defined as a BMI≥25 according to WHO criteria 31 . Interventions were excluded from this analysis if they used exercise forms other than aerobic or resistance exercise (e.g., yoga or Pilates) or were combined with adjuvant therapy. Following these criteria, guidelines from Germany 7 , Europe 32 and the US 9 10 were searched for eligible meta-analyses.

### Measures

For the assessment of reporting quality, the two checklists TIDieR 29 and CERT 30 were used. TIDieR was published as an expansion on existing guidelines (SPIRIT, CONSORT) 28 33 for reporting in RCTs and observational studies. It provides 12 items that focus on the intervention part of a trial ( Table 1 ).

**Table:** **Table 1** TIDieR items.

Item #	Item name	Description
1	BRIEF NAME	Provide the name or a phrase that describes the intervention.
2	WHY	Describe any rationale, theory, or goal of the elements essential to the intervention.
3	WHAT: materials	Describe any physical or informational materials used in the intervention, including those provided to participants or used in intervention delivery or in training of intervention providers. Provide information on where the materials can be assessed (e.g. online appendix, URL).
4	WHAT: procedures	Describe each of the procedures, activities, and/or processes used in the intervention, including any enabling or support activities.
5	WHO PROVIDED	For each category of intervention provider (e.g., psychologist, nursing assistant), describe their expertise, background and any specific training given.
6	HOW	Describe the modes of delivery (e.g., face-to-face or by some other mechanism, such as internet or telephone) of the intervention and whether it was provided individually or in a group.
7	WHERE	Describe the type(s) of location(s) where the intervention occurred, including any necessary infrastructure or relevant features.
8	WHEN and HOW MUCH	Describe the number of times the intervention was delivered and over what period of time including the number of sessions, their schedule, and their duration, intensity or dose.
9	TAILORING	If the intervention was planned to be personalized, titrated or adapted, then describe what, why, when, and how.
10	MODIFICATIONS	If the intervention was modified during the course of the study, describe the changes (what, why, when, and how).
11	HOW WELL: planned	If intervention adherence or fidelity was assessed, describe how and by whom, and if any strategies were used to maintain or improve fidelity, describe them.
12	HOW WELL: actual	If intervention adherence or fidelity was assessed, describe the extent to which the intervention was delivered as planned.

CERT aims to extend TIDieR especially for exercise interventions and consists of 19 items ( Table 2 ). Using both checklists can provide information about whether an exercise-specific guideline like CERT could improve intervention reporting in comparison to TIDieR.

**Table:** **Table 2** CERT items.

Item #	Item name	Description
1	WHAT: materials	Detailed description of the type of exercise equipment (e.g., weights, exercise equipment such as machines, treadmill, bicycle ergometer, etc).
2	WHO: provider	Detailed descriptions of the qualifications, teaching/supervising expertise, and/or training undertaken by the exercise instructor.
3	HOW: delivery	Describe whether exercises are performed individually or in a group.
4		Describe whether exercises are supervised or unsupervised and how they are delivered.
5		Detailed description of how adherence to exercise is measured and reported.
6		Detailed description of motivation strategies.
7a		Detailed description of the decision rule(s) for determining exercise progression.
7b		Detailed description of how the exercise program was progressed.
8		Detailed description of each exercise to enable replication (e.g., photographs, illustrations, video, etc).
9		Detailed description of any home program component (e.g., other exercises, stretching etc).
10		Describe whether there are any non-exercise components (e.g. education, cognitive behavioral therapy, massage, etc).
11		Describe the type and number of adverse events that occurred during exercise.
12	WHERE: location	Describe the setting in which the exercises are performed.
13	WHEN, HOW MUCH: dosage	Detailed description of the exercise intervention including, but not limited to, number of exercise repetitions/sets/sessions, session duration, intervention/program duration, etc.
14a	TAILORING:	
what, how	Describe whether the exercises are generic (one size fits all) or tailored to the individual.	
14b		Detailed description of how exercises are tailored to the individual.
15		Describe the decision rule for determining the starting level at which people commence an exercise program (such as beginner, intermediate, advanced, etc).
16a	HOW WELL: planned, actual	Describe how adherence or fidelity to the exercise intervention is assessed/measured.
16b		Describe the extent to which the intervention was delivered as planned.

Both checklists are not only considered as a guideline for the reporting process but also as a tool for assessing reporting quality.

### Data extraction

Reporting quality of the studies was independently assessed by two reviewers (RJ, BC), who were trained in the application of TIDieR and CERT. Each item was rated with “1,” “0,” or “not applicable,” where “1” marks the item as sufficiently described and “0” as insufficiently. Disagreements were resolved by discussion or by involving a third reviewer (WB). The individual ratings of the two reviewers can be seen in the Supplementary Digital Material, Tables 1 2 3 4 . A percentage score was determined for the completed items of each study as well as for completion of each individual item across all studies.

**Table:** **Table 3** Extraction of the F.I.T.T. principle components. Intensity is defined according to basic literature 42 . As most studies worked with more than one exercise modality, there is a higher number of intervention modalities than studies.

	Frequency	Intensity	Time (program)	Time (exercise)	Type
AE	2×2/week	6x light		1x<=15 min	44x (treadmill) walking
	30×3/week	33x moderate		10x<=30 min	22x (treadmill) running
	9×4/week	11x high		20x<=45 min	22x (ergometer) cycling
	14×5/week	6x n/a		15x<=60 min	11x stair stepping
	1×7/week			3x<=90 min	6x (step) aerobics
	1x n/a		20×1–3 months	3×300 kcal	1 or 2x aqua jogging, circuit training, swimming, gym workout, line dancing, ski ergometer, stretching, light calisthenics, n/a
			12×4–6 months	1×700 kcal	
			4×7–9 months	1×3 miles	
			9×10–12 months	1x n/a	
RE	1×1/week	5x light	1×13–24 months	1×4 sets, 5–7 rpt.	6x free weights
	36×3/week	19x moderate	1x until BMI<25	4×1 set, 8–12 rpt.	26x exercise machines
	1×4/week	2x high		2×2 sets, 8–12 rpt.	17x calisthenics
	1×6/week	3x n/a		12×3 sets, 8–12 rpt.	1x elastic bands
	1x n/a			3×4 sets, 8–12 rpt.	
				1×1 set, 13–15 rpt.	
				1×2 sets, 13–15 rpt.	
				1x n/a	

AE, aerobic exercise; RE, resistance exercise.

**Table:** **Table 4** Interrater agreement of the TIDieR items.

Item	Agreement (%)	PABAK
1	100%	1
2	100%	1
3	66%	0.32
4	68%	0.36
5	57%	0.15
6	66%	0.32
7	62%	0.23
8	36%	– 0.28
9	28%	– 0.45
10	100%	1
11	51%	0.02
12	70%	0.36
Overall	67%	0.34
Core items	55%	0.09

Additionally, for further judgement of intervention description completeness, the F.I.T.T. (frequency, intensity, time, type) principle components were collected. These were developed by the American College of Sports Medicine (ACSM) as a tool for exercise description 34 35 . Following this, frequency, intensity, time, and type are the components that need to be reported for a sufficient endurance or resistance exercise prescription. Furthermore, the respective Journal Impact Factor was determined for each study. If not available (particularly for older studies), the closest available Journal Impact Factor was used.

### Rater agreement

To determine the interrater reliability, for each item the percentage agreement and prevalence-adjusted bias-adjusted kappa (PABAK) were assessed. For this calculation, the individual results of the two raters before the final version were used.

Cohen’s kappa is a coefficient for interrater agreement, which is criticized for depending too heavily on prevalence 36 37 . Therefore, a prevalence-adjusted and bias-adjusted kappa was developed following the formula: PABAK=2 I _0_ –1, where I _0_ is the observed agreement 38 .

For percentage agreement, a score of 80% or higher was considered as acceptable 39 . For PABAK, the strength of agreement is defined as followed:<0.00=poor, 0.00–0.20 slight, 0.21–0.40=fair, 0.41–0.60=moderate, 0.61–0.80=substantial, 0.81–1.00=excellent 40 .

### Data analyses

The individual items of TIDieR and CERT were extracted into Microsoft Excel. They were described by the proportions of sufficiently described items, their means (M), standard deviations (SD), and 95% confidence intervals (95% CI). If an item was rated as “not applicable,” it was treated as a given item, i.e., “yes”. This applied to all studies regarding TIDieR item 10 (modification) and no other items, resulting in a 100% score for this item.

To estimate a potential difference between exercise modalities, studies were divided into two groups. The first group consisted of studies that described only aerobic exercise (AE) and the second consisted of studies that described both aerobic and resistance exercise (ARE). Only CERT was used for this part of the analysis, as this checklist was designed especially for exercise interventions. For a χ ^2^ analysis, only items with a difference in reporting quality between AE and ARE higher than the pooled SD of the overall results of the respective item were included. A Bonferroni correction to the significance level was applied. Hence, the significance level for a single test was divided through the number of tests run on the same data (n=20), which lead to α=0.0025.

Furthermore, the associations between reporting quality (measured as percentage of sufficiently described items of an intervention), year of publication, and Journal Impact Factor were assessed using a Spearman correlation. Correlations and PABAK calculations were made with SPSS Statistics 23 (IBM, Armonk, NY, USA). Statistical significance was set as α=0.05.

## Results

### Included studies

**Fig. 1:**
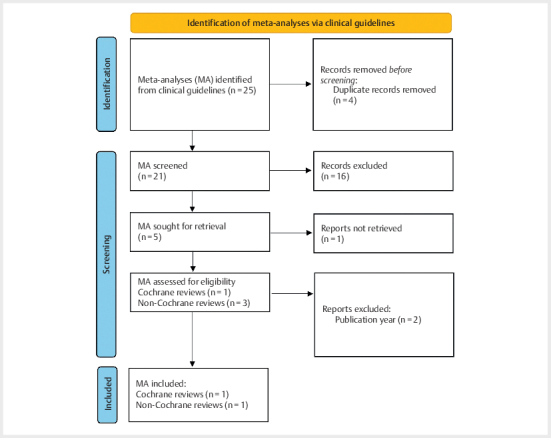
Flow diagram of selection of meta-analyses according to PRISMA 2020 statement 43 .

A total of 25 potential meta-analyses was detected, of which 17 were eligible after removal of duplicates. Of those, four fit with the criteria of population and intervention, including one Cochrane Review 41 and three non-Cochrane meta-analyses. One of each was included so that the most up-to-date meta-analysis according to publication year was chosen 42 . The process of selection is illustrated in the flow diagram ( Fig. 1 ).

A total of 47 studies with 3 474 participants were included (Supplementary Digital Material, Supplementary Text 1: References of the primary studies used in this study). All of them are written in English. Publication years range from 1987 to 2012 with a median of 1996 and an interquartile range (IQR) of 6 (1992 to 1998). The median duration of the exercise program was 16 weeks with a range from 10 to 104 and an IQR of 20 (12 to 32). Table 3 shows an overview of all F.I.T.T. principle components. Regarding exercise modality, n=24 (AE) of all primary studies provided aerobic exercise, and n=23 (ARE) provided a combination of aerobic and resistance exercise. No study provided resistance exercise only.

Extraction of the F.I.T.T. principle components. Intensity is defined according to basic literature [44]. As most studies worked with more than one exercise modality, there is a higher number of intervention modalities than studies.

### Reporting Quality

**Fig. 2:**
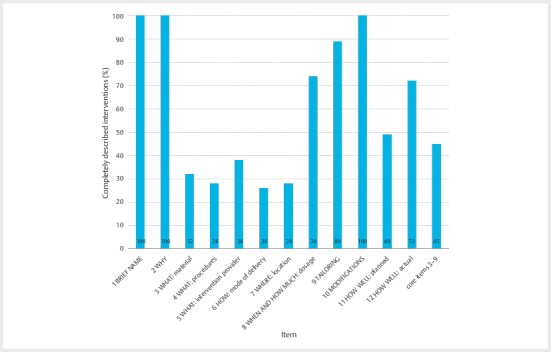
TIDier results.

On average, 7 out of 12 Items of the TIDieR checklist were sufficiently described, resulting in an overall reporting quality of 61% (95% CI: 57.9%–64.8%, min.: 33.3%, max.: 83.3%). For the most essential items required for intervention replication (core items 3–9) 3 out of 7 or 45% (95% CI: 40.0%–49.9%, min.: 0%, max.: 85.7%) were completely reported. Within the core items, the highest score was for item 9 (89%) and the lowest for item 6 (26%). Fig. 2 shows the overall results for each item.

The overall percentage agreement between the two raters was 67% with a PABAK of .34. Considering only the core items, agreement was 55% and PABAK 0.09. Table 4 shows the results for each item on the TIDieR checklist.

**Fig. 3:**
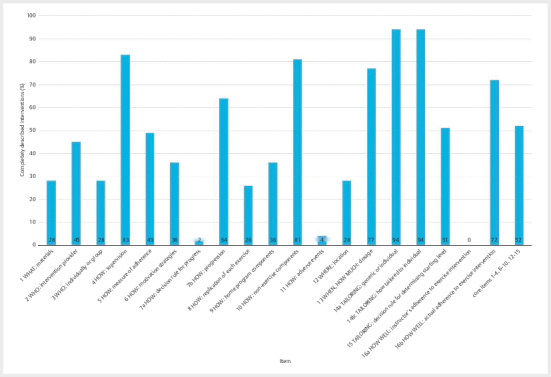
CERT results.

From the CERT checklist, 9 out of 19 items or 47% (95% CI: 43.7%–50.8%, min.: 15.8%, max.: 68.4%) were sufficiently reported. The results for the core items of CERT are 8 out of 15 or 52% (95% CI: 47.6%–55.4%, min.: 20.0%, max.: 80.0%). Within the core items the highest score was for items 14a and 14b (each 94%) and the lowest for item 7a (2%). Fig. 3 shows the results for the individual items.

The percentage agreement between the two raters for all CERT items was 78% with a PABAK of 0.57. For only the core items (items 1–4, 6–10, 12–15) agreement is 77% and PABAK .53.

Table 5 shows the results for the individual items.

**Table:** **Table 5** Interrater agreement of the CERT items.

Item	Agreement (%)	PABAK
1	57%	0.15
2	87%	0.75
3	83%	0.66
4	68%	0.36
5	77%	0.53
6	70%	0.4
7a	81%	0.62
7b	77%	0.53
8	70%	0.4
9	77%	0.53
10	77%	0.53
11	96%	0.92
12	89%	0.79
13	85%	0.7
14a	89%	0.79
14b	91%	0.83
15	45%	–0.11
16a	100%	1
16b	68%	0.36
Overall	78%	0.57
Core items	76%	0.53

All four F.I.T.T. principle components were sufficiently described in 83% of all studies (39 out of 47). Frequency was sufficiently described in 100% of the studies (47 out of 47), intensity in 85% (40 out of 47), time in 98% (46 out of 47), and type in 96% (45 out of 47).

### Difference in completeness of reporting between aerobic and resistance exercise interventions

**Fig. 4:**
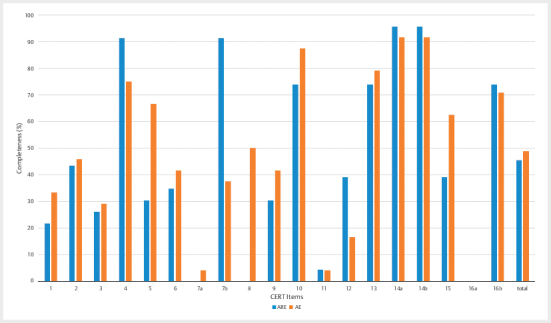
Comparison of AE and ARE results.

On average, AE (n=24) completed 49% of all items of CERT (9.3 out of 19), whereas ARE (n=23) completed 46% (8.7 out of 19). A χ ^2^ analysis showed no significant difference in reporting quality between these groups (χ ^2^ =12.0, p=0.29). Looking at the individual items, two of them (7b and 8) showed a difference in reporting quality higher than the pooled SD. Item 7b (detailed description of how the exercise was progressed) was described sufficiently in 38% of the studies in AE and in 91% of ARE. Item 8 (detailed description of each exercise to enable replication) was sufficiently described in 50% of AE studies and in no study of ARE. For both items, χ ^2^ analysis showed a significant difference (7b: χ ^2^ =14.7, p<0.01; 8: χ ^2^ =15.4, p<0.01). Results for all items can be seen in Fig. 4 .

To further examine potential reasons for these differences, a *t* -test with publication year of AE (M±SD=1996±5.2) and ARE (M±SD=2003±6.6) was calculated and showed a significant difference ( *t* =4.16, p<0.01).

### Association with publication year or Journal Impact Factor

The spearman correlation analysis of reporting quality and publication year or Journal Impact Factor showed no significant results for either TIDieR or CERT. When using only the core items of the checklists, a positive correlation of ρ=0.27 (p=0.04) between CERT and Journal Impact Factor was detected ( Table 6 ).

**Table:** **Table 6** Spearman correlation between reporting quality and publication year/Journal Impact Factor.

	TIDieR	TIDieR core	CERT	CERT core
Publication year	ρ=–0.01	ρ=0.19	ρ=–0.07	ρ=0.04
Journal Impact Factor	ρ=0.13	ρ=0.24	ρ=0.22	ρ=0.27*

^*^ Significant result (≤0.05).

## Discussion

### Reporting quality

Overall, reporting quality of the examined 47 studies can be described as poor. Using the TIDieR checklist, 61% of all items or 45% of the core items were sufficiently reported. The better reporting for all items can be explained by the first two items, which are easily fulfilled, and thus were fully described in all studies. For CERT, the scores are 47% for all and 52% for the core items.

Thus, half of the necessary information is missing, and even though there are no clear criteria on what percentage can be considered sufficient, this lack of essential information can clearly be considered insufficient. Therefore, a replication of exercise interventions for the treatment of overweight in clinical practice is hardly possible. Clinicians depend on more detailed descriptions to ensure a comparable outcome in their treatment interventions or recommendations.

Most of the included studies are older than guidelines like TIDieR and CERT. The discourse within the research community has developed over time, which may provide one possible explanation for the lack of information. Conventions were simply not as clear as they are now.

Other studies have evaluated reporting of exercise interventions for different diseases, e.g., coronary heart disease, peripheral arterial disease, hypertension, osteoporosis, and groin pain/injuries 16 17 18 19 20 21 22 23 24 25 26 using TIDieR and/or CERT. Their results show mostly scores around 50% with few outliers. In comparison, the scores of this study at 61% for TIDieR and 47% for CERT are very similar. Thus, it can be assumed that poor reporting quality is not only a problem of studies with exercise interventions on overweight but also of exercise intervention studies in general for the treatment of several other diseases.

The results of this study also show no substantial differences in reporting quality at least when comparing the overall results of the two checklists TIDieR and CERT. Nevertheless, CERT includes more items asking for more exercise-specific details than TIDieR can cover as a non-specific checklist. To provide as many details as possible in exercise intervention reporting, CERT can accordingly be recommended as a checklist for both authors and editors.

Regarding the most essential exercise components, 83% of all studies reported sufficiently on all four F.I.T.T. components frequency, intensity, time, and type. This shows that, overall, at least the fundamental exercise dosage criteria were reported adequately in the majority of all studies, which provides the foundation for very basic clinical recommendations.

### Difference between aerobic and resistance exercise

Comparing those studies describing only aerobic exercise (AE) with those describing both aerobic and resistance exercise (ARE), two items of the CERT guideline (7b and 8) show a significant difference in reporting quality between both groups.

For Item 7b (detailed description of how the exercise was progressed), ARE has a higher score than AE. One possible explanation might be that for resistance exercise, it could appear more natural for people to progress exercise systematically than for endurance exercise. When writing their papers, authors of ARE studies thus may more likely have thought of reporting exercise progress than authors of AE studies. Another possible explanation for this difference could be higher awareness of reporting standards over time as the *t* -test showed that AE studies were significantly older than ARE studies.

No single study of ARE fulfilled item 8 (detailed description of each exercise to enable replication) adequately. The most important reason for poor resistance exercise description is that these exercises often require such precise movement that only naming them will lead to misunderstandings and thus to wrong movement patterns. In contrast, aerobic exercise typically involves movements like running, walking or cycling. These are easy to understand and do not need any further explanation. Lack of space in the final paper might be a factor for the limited resistance exercise description.

In contrast to our results, an analysis of reporting quality of exercise cancer trials 22 did not find better exercise description of aerobic exercise interventions compared to interventions including resistance exercise. The latter even provided the best results in that study. As the type of population cannot explain these differing results, the authors of this study may have interpreted the item for exercise description (TIDieR item 4) differently than the authors of the cancer trial study. This shows that a precise item description is very important for consistent interpretation and the improvement of reporting quality. As they used only TIDieR, there was no item adequate for CERT item 7b. Therefore, a comparison was not possible.

The F.I.T.T. principle components are key data that need to be given regardless of which type of exercise is done, although there is a difference between modalities in how much information is required, similar to CERT item 8. For example, aerobic exercise like running or cycling does not need further explanation in regard to movement execution. In contrast, individual resistance exercises require more detailed descriptions of movement. This aspect should also be considered in reporting guidelines.

### Interrater agreement

Overall agreement between the raters was 67% (PABAK 0.34) for TIDieR and 78% (PABAK 0.57) for CERT. Considering the first two items of TIDieR are described in all cases, it might be more conclusive in this case to use the interrater agreement for the core items. An agreement of 55% (PABAK 0.09) shows that there was a severe influence of the items with 100% compliance (items 1, 2, and 10). For CERT, a consideration of the core items does not change the result crucially (77%, PABAK 0.53). Following the classification, the percentage agreement for CERT is acceptable and for TIDieR even below. The PABAK ratings show similar results with an agreement between slight and fair for TIDieR and a moderate agreement for CERT.

Some items like TIDieR items 8 (36%, PABAK – 0.28) and 9 (28%, PABAK – 0.45) even show an interrater agreement that can be considered lower than coincidental. Items were understood differently by the raters, which led to these systematical differences. This shows the importance of precise and distinct wording of the items and their requirements.

In summary, the TIDieR scores barely exceed coincidental agreement, whereas the CERT scores show a moderately good agreement. The differences between the two checklists may be due to different item selections and descriptions. Considering that TIDieR item 10 was rated as N/A, and thus as yes for all studies, the lack of information is even more severe. This item asks for information on possible intervention modifications. The issue is that if modifications had been made but not mentioned, we would not know of it, making it impossible to classify the item as unfulfilled. Accordingly, item 10 is not a helpful tool to assess reporting quality.

Additionally, interrater agreement was overall higher for TIDieR than for CERT. The most important factor for this difference is the inclusion of items 1, 2, and 10 in TIDieR, which all resulted in full agreement. This can be explained by how simple and easy to understand and fulfill these items are.

In general, the results obtained cannot be considered satisfying. One possible factor might be the item description. Ambiguous descriptions might lead to different results of the individual raters and thus should be formulated clearly and precisely.

### Association of reporting quality with publication year and Journal Impact Factor

There was no association found between publication year and reporting quality as in most other studies 19 24 45 46 47 48 . Only Hacke et al. 18 and Thabane 27 found indications of a possible association. A better awareness of reporting standards due to checklists like TIDieR and CERT may be assumed, which would lead to an improvement in reporting quality over time. Additionally, enhancement of technological possibilities may be another factor for the improvement of reporting quality. For a few years now, authors can offer online supplementary material as articles typically have a word limit. For example, implementing a detailed description of exercises would easily be possible using a supplementary material section. Unfortunately, none of the analyzed studies took advantage of such possibilities. On the other hand, most studies analyzed in the present study are too old and did not offer these possibilities at the time of publication.

At the time of data acquisition, the most recent clinical guidelines were used to find possible meta-analyses for inclusion in this study. Even though newer ones may exist, they had not yet been used in clinical guidelines and it was important to the authors to use only meta-analyses that had an impact on clinical practice through guidelines.

A positive correlation between Journal Impact Factor and reporting quality appeared only when using the CERT core items (ρ=0.27, p=0.04). The assumption that journals with a higher Journal Impact Factor focus more on reporting quality than journals with a lower Journal Impact Factor might be expected. Nevertheless, a strong association was not demonstrated in the present study, especially as there are no significant results when using all item scores. Other studies support the assumption that there is no close association between Journal Impact Factor and report quality 18 19 24 48 .

Considering the small sample size for the Spearman correlation, further research with an appropriate number of studies might lead to more conclusive results.

In this analysis, the impact of the individual studies was only controlled through their implementation in meta-analyses that were used in clinical guidelines. For future research, the association with their citations could lead to better insights in this regard.

### Strengths and limitations

The strength of this study is that, to our knowledge, it is the first to identify reporting quality of exercise intervention trials for the treatment of overweight using internationally accepted intervention description guidelines. The serious lack of information shows the need for further systematic assessments of intervention description quality not only for obesity but also for internal diseases in general 24 .

Additionally, before this study there was only one other examining the difference in reporting quality between aerobic and resistance exercise studies. The results show the need for further research as well as the improvement and dissemination of guidelines.

As a limitation of this study, the slightly (TIDieR) to moderate (CERT) PABAK scores show the high level of subjectivity in the assessment of intervention reporting quality with reporting guidelines. Different interpretations of guideline items can lead to widely differing results. Thus, the results of this study must be viewed with respect to interrater variability. A more detailed and distinct description of items might lead to stronger agreement between raters in future. Then again, the lack of item completeness in this study is strong enough to clearly state poor reporting quality.

For future reporting on exercise interventions, we recommend using CERT as it provides more specific guidance. Furthermore, journals should use the guideline as a tool for checking on reporting quality of submitted articles. A high standard of intervention reporting quality will lead to improvement in intervention and research quality in general.

### Implications for practice and further research

The poor reporting quality might lead to uncertainty about exercise intervention modalities. Even though the F.I.T.T. components were mostly reported adequately, half of all necessary information is nevertheless missing. Intervention circumstances as described in TIDieR and CERT need to be better reported so clinicians and researchers can improve their own interventions based on these details or make appropriate recommendations.

Especially clinical guidelines will benefit from improved reporting on exercise interventions. Currently only an implementation of physical activity of at least 150 min/week is recommended 7 8 9 10 . This mirrors the insufficient intervention descriptions describing mostly the core components of exercise dosage (F.I.T.T.) but not much more. To provide a strong and detailed base for exercise recommendations, intervention descriptions need to improve.

Finally, it might be helpful to think about the implementation of disease-specific reporting criteria 24 (24). For exercise interventions in the treatment of breast cancer, Bünzen et al. 25 used CERT as a base for the CORE-CERT checklist, which can serve as an example in developing such checklists for other diagnoses. In the treatment of overweight, a negative energy balance will lead to weight loss 10 . Accordingly, reporting by clinical studies should include information about how energy intake and expenditure were estimated and recorded. Nevertheless, a thorough estimation of targeted and actual caloric intake and expenditure is not yet covered by the existing CERT items. Although these disease-specific items are not included in CERT, it currently provides the best existing tool for exercise intervention reporting.

## Conclusion

In conclusion, the quality of reporting of exercise interventions in the treatment of overweight was found to be insufficient in both AE and ARE studies. For clinical practice and further research, better reporting quality is absolutely necessary in the future. For this purpose, we recommend the usage of CERT as a guideline to authors for reporting on exercise interventions as well as to journal editors to ensure completeness of submitted articles.

Low interrater reliability demonstrates the need to improve some items. Especially item 8 (detailed description of each exercise to enable replication) stood out with differences between the raters as it might be understood ambiguously regarding resistance exercise.

In general, there is evidence for differences in reporting quality between exercise modalities (aerobic vs. resistance exercise). Possible factors might be disparities between exercise modalities in which information is commonly linked with them (e.g., exercise progress may be more commonly linked with resistance than with aerobic exercise), and ambiguous item descriptions. Further research is needed to better explain such differences. Finally, a revision of CERT might be helpful to reduce uncertainties.
