# Supplementary material for: Aerobic Versus Resistance Exercise for Overweight: Is there a Difference in Reporting Quality?
Source: Sports Med Int Open. 2025 Jul 28;9:a25962049. doi: 10.1055/a-2596-2049 (PMC12372422; doi:10.1055/a-2596-2049)
Supplement: Supplementary file 1 — Supplementary Material [file 10-1055-a-2596-2049-11-2024-0260-re.pdf]

Supplementary Digital Material

Supplementary Text 1: References of the primary studies used in this study.

[1] Ahmadizad S, Haghighi AH, Hamedinia MR. Effects of resistance versus endurance training on serum adiponectin and insulin resistance index. Eur J Endocrinol 2007; 157: 625–631. doi:10.1530/EJE-07-0223

[2] Andersen RE, Wadden TA, Bartlett SJ, et al. Effects of lifestyle activity vs structured aerobic exercise in obese women: a randomized trial. JAMA 1999; 281: 335–340. doi:10.1001/jama.281.4.335

[3] Anderssen SA, Hjermann I, Urdal P, et al. Improved carbohydrate metabolism after physical training and dietary intervention in individuals with the "atherothrombogenic syndrome'. Oslo Diet and Exercise Study (ODES). A randomized trial. J Intern Med 1996; 240: 203–209. doi:10.1046/j.1365-2796.1996.22848000.x

[4] Ballor DL, Harvey-Berino JR, Ades PA, et al. Contrasting effects of resistance and aerobic training on body composition and metabolism after diet-induced weight loss. Metabolism 1996; 45: 179–183. doi:10.1016/s0026-0495(96)90050-5

[5] Banz WJ, Maher MA, Thompson WG, et al. Effects of resistance versus aerobic training on coronary artery disease risk factors. Exp Biol Med (Maywood) 2003; 228: 434–440. doi:10.1177/153537020322800414

[6] Bateman LA, Slentz CA, Willis LH, et al. Comparison of aerobic versus resistance exercise training effects on metabolic syndrome (from the Studies of a Targeted Risk Reduction Intervention Through Defined Exercise - STRRIDE-AT/RT). Am J Cardiol 2011; 108: 838–844. doi:10.1016/j.amjcard.2011.04.037

[7] Cox KL, Burke V, Morton AR, et al. Independent and additive effects of energy restriction and exercise on glucose and insulin concentrations in sedentary overweight men. Am J Clin Nutr 2004; 80: 308–316. doi:10.1093/ajcn/80.2.308

[8] Davidson LE, Hudson R, Kilpatrick K, et al. Effects of exercise modality on insulin resistance and functional limitation in older adults: a randomized controlled trial. Arch Intern Med 2009; 169: 122–131. doi:10.1001/archinternmed.2008.558

[9] Donges CE, Duffield R, Drinkwater EJ. Effects of resistance or aerobic exercise training on interleukin-6, C-reactive protein, and body composition. Med Sci Sports Exerc 2010; 42: 304–313. doi:10.1249/MSS.0b013e3181b117ca

[10] Fenkci S, Sarsan A, Rota S, et al. Effects of resistance or aerobic exercises on metabolic parameters in obese women who are not on a diet. Adv Ther 2006; 23: 404–413. doi:10.1007/BF02850161

[11] Fisher G, Hyatt TC, Hunter GR, et al. Effect of diet with and without exercise training on markers of inflammation and fat distribution in overweight women. Obesity (Silver Spring) 2011; 19: 1131–1136. doi:10.1038/oby.2010.310

[12] Gordon NF, Scott CB, Levine BD. Comparison of single versus multiple lifestyle interventions: are the antihypertensive effects of exercise training and diet-induced weight loss additive? Am J Cardiol 1997; 79: 763–767. doi:10.1016/s0002-9149(96)00864-8

[13] Hays NP, Starling RD, Liu X, et al. Effects of an ad libitum low-fat, high-carbohydrate diet on body weight, body composition, and fat distribution in older men and women: a randomized controlled trial. Arch Intern Med 2004; 164: 210–217. doi:10.1001/archinte.164.2.210

Sports Medicine International Open | DOI 10.1055/a-2596-2049 | © 2025. The Author(s).

[14] Hellénius ML, Faire U de, Berglund B, et al. Diet and exercise are equally effective in reducing risk for cardiovascular disease. Results of a randomized controlled study in men with slightly to moderately raised cardiovascular risk factors. Atherosclerosis 1993; 103: 81–91. doi:10.1016/0021-9150(93)90042-s
[15] Irwin ML, Yasui Y, Ulrich CM, et al. Effect of exercise on total and intra-abdominal body fat in postmenopausal women: a randomized controlled trial. JAMA 2003; 289: 323–330. doi:10.1001/jama.289.3.323
[16] Jakicic JM, Wing RR, Butler BA, et al. Prescribing exercise in multiple short bouts versus one continuous bout: effects on adherence, cardiorespiratory fitness, and weight loss in overweight women. Int J Obes Relat Metab Disord 1995; 19: 893–901
[17] Jakicic JM, Marcus BH, Gallagher KI, et al. Effect of exercise duration and intensity on weight loss in overweight, sedentary women: a randomized trial. JAMA 2003; 290: 1323–1330. doi:10.1001/jama.290.10.1323
[18] Janssen I, Fortier A, Hudson R, et al. Effects of an energy-restrictive diet with or without exercise on abdominal fat, intermuscular fat, and metabolic risk factors in obese women. Diabetes Care 2002; 25: 431–438. doi:10.2337/diacare.25.3.431
[19] Kiernan M, King AC, Stefanick ML, et al. Men gain additional psychological benefits by adding exercise to a weight-loss program. Obes Res 2001; 9: 770–777. doi:10.1038/oby.2001.106
[20] Leutholtz BC, Keyser RE, Heusner WW, et al. Exercise training and severe caloric restriction: effect on lean body mass in the obese. Arch Phys Med Rehabil 1995; 76: 65–70. doi:10.1016/s0003-9993(95)80045-x
[21] Martins RA, Neves AP, Coelho-Silva MJ, et al. The effect of aerobic versus strength-based training on high-sensitivity C-reactive protein in older adults. Eur J Appl Physiol 2010; 110: 161–169. doi:10.1007/s00421-010-1488-5
[22] Neumark-Sztainer D, Kaufmann NA, Berry EM. Physical activity within a community-based weight control program: program evaluation and predictors of success. Public Health Rev 1995; 23: 237–251
[23] Nieman DC, Nehlsen-Cannarella SL, Henson DA, et al. Immune response to exercise training and/or energy restriction in obese women. Med Sci Sports Exerc 1998; 30: 679–686. doi:10.1097/00005768-199805000-00006
[24] Potteiger JA, Claytor RP, Hulver MW, et al. Resistance exercise and aerobic exercise when paired with dietary energy restriction both reduce the clinical components of metabolic syndrome in previously physically inactive males. Eur J Appl Physiol 2012; 112: 2035–2044. doi:10.1007/s00421-011-2174-y
[25] Pritchard JE, Nowson CA, Wark JD. Bone loss accompanying diet-induced or exercise-induced weight loss: a randomised controlled study. Int J Obes Relat Metab Disord 1996; 20: 513–520
[26] Pritchard JE, Nowson CA, Wark JD. A worksite program for overweight middle-aged men achieves lesser weight loss with exercise than with dietary change. J Am Diet Assoc 1997; 97: 37–42. doi:10.1016/S0002-8223(97)00015-1
[27] Rice B, Janssen I, Hudson R, et al. Effects of aerobic or resistance exercise and/or diet on glucose tolerance and plasma insulin levels in obese men. Diabetes Care 1999; 22: 684–691. doi:10.2337/diacare.22.5.684
[28] Ross R, Rissanen J. Mobilization of visceral and subcutaneous adipose tissue in response to energy restriction and exercise. Am J Clin Nutr 1994; 60: 695–703. doi:10.1093/ajcn/60.5.695

[29] Ross R, Rissanen J, Pedwell H, et al. Influence of diet and exercise on skeletal muscle and visceral adipose tissue in men. J Appl Physiol (1985) 1996; 81: 2445–2455. doi:10.1152/jappl.1996.81.6.2445

[30] Sarsan A, Ardiç F, Ozgen M, et al. The effects of aerobic and resistance exercises in obese women. Clin Rehabil 2006; 20: 773–782. doi:10.1177/0269215506070795

[31] Schwartz RS. The independent effects of dietary weight loss and aerobic training on high density lipoproteins and apolipoprotein A-I concentrations in obese men. Metabolism 1987; 36: 165–171. doi:10.1016/0026-0495(87)90012-6

[32] Schwartz RS, Jaeger LF, Veith RC, et al. The effect of diet or exercise on plasma norepinephrine kinetics in moderately obese young men. Int J Obes 1990; 14: 1–11

[33] Stefanick ML, Mackey S, Sheehan M, et al. Effects of diet and exercise in men and postmenopausal women with low levels of HDL cholesterol and high levels of LDL cholesterol. N Engl J Med 1998; 339: 12–20. doi:10.1056/NEJM199807023390103

[34] Stensvold D, Tjønna AE, Skaug E-A, et al. Strength training versus aerobic interval training to modify risk factors of metabolic syndrome. J Appl Physiol (1985) 2010; 108: 804–810. doi:10.1152/japplphysiol.00996.2009

[35] Svendsen OL, Hassager C, Christiansen C. Effect of an energy-restrictive diet, with or without exercise, on lean tissue mass, resting metabolic rate, cardiovascular risk factors, and bone in overweight postmenopausal women. Am J Med 1993; 95: 131–140. doi:10.1016/0002-9343(93)90253-l

[36] Svendsen OL, Krotkiewski M, Hassager C, et al. Effects on muscle of dieting with or without exercise in overweight postmenopausal women. J Appl Physiol (1985) 1996; 80: 1365–1370. doi:10.1152/jappl.1996.80.4.1365

[37] Thong FS, Hudson R, Ross R, et al. Plasma leptin in moderately obese men: independent effects of weight loss and aerobic exercise. Am J Physiol Endocrinol Metab 2000; 279: E307-13. doi:10.1152/ajpendo.2000.279.2.E307

[38] van Aggel-Leijssen DP, Saris WH, Hul GB, et al. Short-term effects of weight loss with or without low-intensity exercise training on fat metabolism in obese men. Am J Clin Nutr 2001; 73: 523–531. doi:10.1093/ajcn/73.3.523

[39] Wadden TA, Vogt RA, Andersen RE, et al. Exercise in the treatment of obesity: effects of four interventions on body composition, resting energy expenditure, appetite, and mood. J Consult Clin Psychol 1997; 65: 269–277. doi:10.1037//0022-006x.65.2.269

[40] Wallace MB, Mills BD, Browning CL. Effects of cross-training on markers of insulin resistance/hyperinsulinemia. Med Sci Sports Exerc 1997; 29: 1170–1175. doi:10.1097/00005768-199709000-00008

[41] Whatley JE, Gillespie WJ, Honig J, et al. Does the amount of endurance exercise in combination with weight training and a very-low-energy diet affect resting metabolic rate and body composition? Am J Clin Nutr 1994; 59: 1088–1092. doi:10.1093/ajcn/59.5.1088

[42] Willis LH, Slentz CA, Bateman LA, et al. Effects of aerobic and/or resistance training on body mass and fat mass in overweight or obese adults. J Appl Physiol (1985) 2012; 113: 1831–1837. doi:10.1152/japplphysiol.01370.2011

[43] Wing RR, Epstein LH, Paternostro-Bayles M, et al. Exercise in a behavioural weight control programme for obese patients with Type 2 (non-insulin-dependent) diabetes. Diabetologia 1988; 31: 902–909. doi:10.1007/BF00265375

[44] Wing RR, Venditti E, Jakicic JM, et al. Lifestyle intervention in overweight individuals with a family history of diabetes. Diabetes Care 1998; 21: 350–359. doi:10.2337/diacare.21.3.350
[45] Wood PD, Stefanick ML, Dreon DM, et al. Changes in plasma lipids and lipoproteins in overweight men during weight loss through dieting as compared with exercise. N Engl J Med 1988; 319: 1173–1179. doi:10.1056/NEJM198811033191801
[46] Wood PD, Stefanick ML, Williams PT, et al. The effects on plasma lipoproteins of a prudent weight-reducing diet, with or without exercise, in overweight men and women. N Engl J Med 1991; 325: 461–466. doi:10.1056/NEJM199108153250703

Supplementary Table 1: TIDieR Results Reviewer 1.

Item / Study	1 BRIEF NAME	2 WHY	3 WHAT: Materials	4 WHAT: Procedures	5 WHO PROVIDED	6 HOW	7 WHERE	8 WHEN AND HOW MUCH	9 TAILORING	10 MODIFICATIONS	11 HOW WELL: Planned	12 HOW WELL: Actual	Core Items (3-9) Total	Core Items (%)	All Items Total	All Items (%)
Ahmadizad et al. 2007	1	1	0	0	0	0	0	1	1	1	0	0	2	28,57	5	41,67
Ballor et al. 1996	1	1	0	0	0	1	0	1	1	1	0	0	3	42,86	6	50,00
Banz et al. 2003	1	1	1	0	0	1	1	1	1	1	1	1	5	71,43	10	83,33
Bateman et al. 2011	1	1	0	0	0	0	0	1	1	1	1	1	2	28,57	7	58,33
Davidson et al. 2009	1	1	0	0	0	0	0	1	1	1	0	1	2	28,57	6	50,00
Donges et al. 2010	1	1	0	0	1	0	1	1	1	1	1	1	4	57,14	9	75,00
Fenkci et al. 2006	1	1	1	0	0	0	0	1	1	1	0	0	3	42,86	6	50,00
Sarsan et al. 2006	1	1	1	0	0	0	1	1	1	1	0	0	4	57,14	7	58,33
Fisher et al. 2011	1	1	0	0	0	0	1	1	1	1	0	0	3	42,86	6	50,00
Janssen et al. 2002	1	1	0	0	1	0	0	1	1	1	0	1	3	42,86	7	58,33
Martins et al. 2010	1	1	1	0	1	0	0	0	0	1	0	0	2	28,57	5	41,67
Potteiger et al. 2012	1	1	0	0	1	1	1	0	1	1	0	1	4	57,14	8	66,67
Rice et al. 1999	1	1	0	0	0	0	0	1	1	1	0	1	2	28,57	6	50,00
Ross et al. 1994	1	1	0	0	0	0	0	1	1	1	0	1	2	28,57	6	50,00
Stensvold et al. 2010	1	1	0	0	1	0	1	1	1	1	0	1	4	57,14	8	66,67
Wallace et al. 1997	1	1	0	0	1	0	0	1	1	1	0	1	3	42,86	7	58,33
Willis et al. 2012	1	1	0	0	0	0	0	1	1	1	1	1	2	28,57	7	58,33
Aggel-Leijsen et al. 2001	1	1	1	1	0	0	1	1	1	1	1	1	5	71,43	10	83,33
Andersen et al. 1999	1	1	1	0	1	1	1	1	1	1	0	1	6	85,71	10	83,33
Anderssen et al. 1996	1	1	0	0	0	0	0	0	1	1	1	1	1	14,29	6	50,00
Cox et al. 2004	1	1	1	0	1	0	0	1	1	1	0	1	4	57,14	8	66,67
Gordon et al. 1997	1	1	0	0	1	0	1	1	1	1	1	0	4	57,14	8	66,67
Hays et al. 2004	1	1	1	1	0	0	0	1	1	1	0	0	4	57,14	7	58,33
Hellenius et al. 1993	1	1	0	1	1	0	0	1	1	1	1	1	4	57,14	9	75,00
Irwin et al. 2003	1	1	0	0	1	1	1	0	1	1	1	1	4	57,14	9	75,00
Jakicic et al. 1995	1	1	0	0	0	0	0	1	1	1	1	1	2	28,57	7	58,33
Jakicic et al. 2003	1	1	1	1	0	0	0	1	1	1	1	1	4	57,14	9	75,00
Kiernan et al. 2001	1	1	0	1	0	0	0	1	1	1	1	0	3	42,86	7	58,33
Leutholtz et al. 1995	1	1	0	1	0	0	0	1	1	1	1	1	3	42,86	8	66,67
Neumark-Sztainer et al. 1995	1	1	0	0	1	0	1	0	0	1	1	1	2	28,57	7	58,33
Nieman et al. 1998	1	1	1	0	0	0	1	0	1	1	1	1	3	42,86	8	66,67
Pritchard et al. 1997	1	1	1	1	0	0	0	1	1	1	1	1	4	57,14	9	75,00
Pritchard et al. 1996	1	1	0	1	0	0	0	1	1	1	1	1	3	42,86	8	66,67
Ross et al. 1996	1	1	0	0	0	0	0	0	0	1	0	1	0	0,00	4	33,33
Schwartz 1987	1	1	0	0	1	0	0	1	1	1	1	1	3	42,86	8	66,67
Schwartz et al. 1990	1	1	0	0	1	0	0	1	1	1	1	1	3	42,86	8	66,67
Stefanick et al. 1998	1	1	0	1	0	1	0	1	1	1	0	0	4	57,14	7	58,33
Svendsen et al. 1993	1	1	0	0	1	1	0	1	1	1	1	1	4	57,14	9	75,00
Svendsen et al. 1996	1	1	0	0	0	0	0	1	1	1	0	1	2	28,57	6	50,00
Thong et al. 2000	1	1	1	1	0	0	0	1	1	1	1	1	4	57,14	9	75,00
Wadden et al. 1997	1	1	0	0	1	1	1	0	1	1	0	1	4	57,14	8	66,67
Whatley et al. 1994	1	1	0	0	1	0	0	1	1	1	0	1	3	42,86	7	58,33
Wing et al. 1988 (1)	1	1	1	0	0	0	0	0	0	1	0	1	1	14,29	5	41,67
Wing et al. 1988 (2)	1	1	1	1	0	1	0	0	0	1	0	1	3	42,86	7	58,33
Wing et al. 1998	1	1	1	1	1	1	0	0	1	1	0	0	5	71,43	8	66,67
Wood et al. 1988	1	1	0	0	0	1	0	0	1	1	1	0	2	28,57	6	50,00
Wood et al. 1991	1	1	0	1	0	1	0	1	1	1	1	0	4	57,14	8	66,67
Described Total	47	47	15	13	18	12	13	35	42	47	23	34	3,15	44,98	7,36	61,35
Described (%)	100,00	100,00	31,91	27,66	38,30	25,53	27,66	74,47	89,36	100,00	48,94	72,34				

Sports Medicine International Open | DOI 10.1055/a-2596-2049 | © 2025. The Author(s).

Supplementary Table 2: TIDieR Results Reviewer 2.

Study \ Item	1 BRIEF NAME	2 WHY	3 WHAT: Materials	4 WHAT: Procedures	5 WHO PROVIDED	6 HOW	7 WHERE	8 WHEN AND HOW MUCH	9 TAILORING	10 MODIFICATIONS	11 HOW WELL: Planned	12 HOW WELL: Actual	Core Items (3-9) Total	Core Items (%)	All Items Total	All Items (%)
Ahmadizad et al. 2007	1	1	0	0	0	0	0	0	0	1	0	0	0	0,00	3	25,00
Ballor et al. 1996	1	1	0	0	0	0	0	0	0	1	0	0	0	0,00	3	25,00
Banz et al. 2003	1	1	0	0	0	0	0	0	0	1	0	0	0	0,00	3	25,00
Bateman et al. 2011	1	1	1	1	1	1	1	1	1	1	0	0	7	100,00	10	83,33
Davidson et al. 2009	1	1	0	0	0	0	0	0	0	1	0	1	0	0,00	4	33,33
Donges et al. 2010	1	1	0	0	0	0	0	0	0	1	0	0	0	0,00	3	25,00
Fenkci et al. 2006	1	1	0	0	0	0	0	0	0	1	0	0	0	0,00	3	25,00
Sarsan et al. 2006	1	1	0	0	0	0	0	0	0	1	0	0	0	0,00	3	25,00
Fisher et al. 2011	1	1	0	0	0	0	0	0	0	1	0		0	0,00	3	25,00
Janssen et al. 2002	1	1	0	0	0	0	0	0	0	1	0	1	0	0,00	4	33,33
Martins et al. 2010	1	1	0	0	0	0	0	0	0	1	0	0	0	0,00	3	25,00
Potteiger et al. 2012	1	1	0	0	0	0	0	0	0	1	0	1	0	0,00	4	33,33
Rice et al. 1999	1	1	0	0	0	0	0	0	0	1	0	1	0	0,00	4	33,33
Ross et al. 1994	1	1	0	0	0	0	0	0	0	1	0	0	0	0,00	3	25,00
Stensvold et al. 2010	1	1	0	0	0	0	0	0	0	1	0	0	0	0,00	3	25,00
Wallace et al. 1997	1	1	0	0	0	0	0	0	0	1	0	1	0	0,00	4	33,33
Willis et al. 2012	1	1	0	0	0	0	0	0	0	1	0	0	0	0,00	3	25,00
Aggel-Leijsen et al. 2001	1	1	0	0	0	0	0	0	0	1	0	0	0	0,00	3	25,00
Andersen et al. 1999	1	1	1	1	1	1	1	1	1	1	0	0	7	100,00	10	83,33
Anderssen et al. 1996	1	1	0	0	0	0	0	0	0	1	0	1	0	0,00	4	33,33
Cox et al. 2004	1	1	0	0	0	0	0	0	0	1	0	0	0	0,00	3	25,00
Gordon et al. 1997	1	1	1	1	1	1	1	1	1	1	0	0	7	100,00	10	83,33
Hays et al. 2004	1	1	0	0	0	0	0	0	0	1	0	0	0	0,00	3	25,00
Hellenius et al. 1993	1	1	0	0	0	0	0	0	0	1	0	0	0	0,00	3	25,00
Irwin et al. 2003	1	1	0	0	0	0	0	0	0	1	0	1	0	0,00	4	33,33
Jakicic et al. 1995	1	1	1	1	1	1	1	1	1	1	0	0	7	100,00	10	83,33
Jakicic et al. 2003	1	1	0	0	0	0	0	0	0	1	0	0	0	0,00	3	25,00
Kiernan et al. 2001	1	1	0	0	0	0	0	0	0	1	0	0	0	0,00	3	25,00
Leutholtz et al. 1995	1	1	0	0	0	0	0	0	0	1	0	1	0	0,00	4	33,33
Neumark-Sztainer et al. 1995	1	1	0	0	0	0	0	0	0	1	0	1	0	0,00	4	33,33
Nieman et al. 1998	1	1	0	0	0	0	0	0	0	1	0	1	0	0,00	4	33,33
Pritchard et al. 1997	1	1	1	1	1	1	1	1	1	1	0	1	7	100,00	11	91,67
Pritchard et al. 1996	1	1	1	1	1	1	1	1	1	1	0	0	7	100,00	10	83,33
Ross et al. 1996	1	1	0	0	0	0	0	0	0	1	0	1	0	0,00	4	33,33
Schwartz 1987	1	1	0	0	0	0	0	0	0	1	0	1	0	0,00	4	33,33
Schwartz et al. 1990	1	1	0	0	0	0	0	0	0	1	0	1	0	0,00	4	33,33
Stefanick et al. 1998	1	1	0	0	0	0	0	0	0	1	0	0	0	0,00	3	25,00
Svendsen et al. 1993	1	1	0	0	0	0	0	0	0	1	0	1	0	0,00	4	33,33
Svendsen et al. 1996	1	1	0	0	0	0	0	0	0	1	0	1	0	0,00	4	33,33
Thong et al. 2000	1	1	1	1	1	1	1	1	1	1	0	1	7	100,00	11	91,67
Wadden et al. 1997	1	1	1	1	1	1	1	1	1	1	0	1	7	100,00	11	91,67
Whatley et al. 1994	1	1	1	1	1	1	1	1	1	1	0	1	7	100,00	11	91,67
Wing et al. 1988 (1)	1	1	1	1	1	1	1	1	1	1	0	0	7	100,00	10	83,33
Wing et al. 1988 (2)	1	1	1	1	1	1	1	1	1	1	0	1	7	100,00	11	91,67
Wing et al. 1998	1	1	1	1	1	1	1	1	1	1	0	1	7	100,00	11	91,67
Wood et al. 1988	1	1	0	0	0	0	0	0	0	1	0	0	0	0,00	3	25,00
Wood et al. 1991	1	1	0	0	0	0	0	0	0	1	0	0	0	0,00	3	25,00
Described Total	47	47	12	12	12	12	12	12	12	47	0	21	1,79	25,53	5,23	43,62
Described (%)	100,00	100,00	25,53	25,53	25,53	25,53	25,53	25,53	25,53	100,00	0,00	44,68				

Sports Medicine International Open | DOI 10.1055/a-2596-2049 | © 2025. The Author(s).

Supplementary Table 3: CERT Results Reviewer 1.

Item / Study	1 WHAT (Material)	2 WHO: provider	3 HOW: delivery (individual or group)	4 HOW: delivery (supervision)	5 HOW: delivery (report and measure of adherence)	6 HOW: delivery (motivation strategies)	7a HOW: delivery (progression rules)	7b HOW: delivery (progression description)	8 HOW: delivery (exercise description)	9 HOW: delivery (home program)	10 HOW: delivery (non-exercise components)	11 HOW: delivery (adverse events)	12 WHERE: location	13 WHEN, HOW MUCH: dosage	14a TAILORING: what	14b TAILORING: how	15 TAILORING: starting level	16a HOW WELL: planned	16b HOW WELL: actual	Core Items (1-4, 6-10, 12-15) Total	Core Items (%)	All Items Total	All Items (%)
Ahmadizad et al. 2007	0	0	0	0	0	0	0	0	0	0	0	0	1	1	1	1	0	0	0	4	26,67	4	21,05
Ballor et al. 1996	1	0	0	0	0	0	1	1	0	0	0	0	0	1	1	1	0	0	0	6	40,00	6	31,58
Banz et al. 2003	1	0	1	1	0	0	0	1	0	0	0	0	0	1	1	1	0	0	0	7	46,67	7	36,84
Bateman et al. 2011	1	0	0	0	1	0	0	1	0	0	0	0	0	1	1	1	0	0	0	5	33,33	6	31,58
Davidson et al. 2009	0	0	0	0	0	0	0	0	0	0	1	0	0	1	1	1	0	0	1	4	26,67	5	26,32
Donges et al. 2010	1	1	0	1	0	0	0	0	0	0	0	0	1	1	1	1	0	0	1	7	46,67	8	42,11
Fenkci et al. 2006	1	0	0	0	0	0	0	1	0	0	0	0	0	1	1	1	1	0	0	6	40,00	6	31,58
Sarsan et al. 2006	1	0	0	0	0	0	1	1	0	0	0	0	0	1	1	1	1	0	0	7	46,67	7	36,84
Fisher et al. 2011	1	0	0	1	0	0	1	1	0	0	1	0	1	1	1	1	0	0	0	9	60,00	9	47,37
Janssen et al. 2002	1	1	0	1	0	0	0	1	0	0	1	0	0	1	1	1	0	0	1	8	53,33	9	47,37
Martins et al. 2010	0	1	0	1	0	0	1	1	0	0	1	0	0	1	1	1	0	0	0	8	53,33	8	42,11
Potteiger et al. 2012	1	1	1	1	0	0	0	1	0	0	1	0	1	1	1	1	0	0	1	10	66,67	11	57,89
Rice et al. 1999	1	0	0	1	1	0	0	0	0	0	1	0	0	1	1	1	0	0	0	6	40,00	7	36,84
Ross et al. 1994	1	0	0	0	0	0	0	1	0	0	1	0	0	1	1	1	0	0	0	6	40,00	6	31,58
Stensvold et al. 2010	1	0	0	1	0	0	1	1	0	0	0	0	1	1	1	1	0	0	0	8	53,33	8	42,11
Wallace et al. 1997	1	1	0	1	0	0	0	1	0	0	1	0	0	1	1	1	0	0	0	8	53,33	8	42,11
Willis et al. 2012	1	0	0	1	1	0	0	1	0	0	1	0	0	1	1	1	0	0	0	7	46,67	8	42,11
Aggel-Leijsen et al. 2001	1	0	0	1	1	0	0	0	1	1	1	0	1	1	1	1	0	0	0	9	60,00	10	52,63
Andersen et al. 1999	1	0	0	1	1	0	0	1	1	0	1	0	1	1	1	1	1	0	0	10	66,67	11	57,89
Anderssen et al. 1996	0	0	0	0	1	0	0	0	0	0	1	0	0	1	1	1	0	0	1	4	26,67	6	31,58
Cox et al. 2004	1	0	0	1	1	1	0	0	0	0	1	0	0	1	1	1	0	0	0	7	46,67	8	42,11
Gordon et al. 1997	0	0	0	0	1	0	0	0	0	0	1	1	1	1	1	1	0	0	0	5	33,33	7	36,84
Hays et al. 2004	1	0	0	0	0	0	0	0	0	0	1	0	0	1	1	1	0	0	0	5	33,33	5	26,32
Hellenius et al. 1993	0	1	0	1	0	0	0	0	0	0	1	0	0	1	1	1	0	0	0	6	40,00	6	31,58
Irwin et al. 2003	1	0	0	0	1	0	0	1	0	1	0	0	1	1	1	1	0	0	1	7	46,67	9	47,37
Jakicic et al. 1995	0	0	0	0	1	0	0	0	0	0	1	0	0	1	1	1	0	0	1	4	26,67	6	31,58
Jakicic et al. 2003	1	0	0	1	1	0	0	0	0	0	0	0	0	1	1	1	0	0	1	5	33,33	7	36,84
Kiernan et al. 2001	0	0	0	1	0	0	1	1	0	0	1	0	0	1	1	1	0	0	0	7	46,67	7	36,84
Leutholtz et al. 1995	0	0	0	0	1	0	0	0	0	0	1	0	0	1	1	1	0	0	1	4	26,67	6	31,58
Neumark-Sztainer et al. 1995	0	1	0	1	1	0	0	0	0	0	1	0	0	0	0	0	0	0	1	3	20,00	5	26,32
Nieman et al. 1998	0	0	0	1	1	0	1	1	0	0	1	0	1	1	1	1	0	0	1	8	53,33	10	52,63
Pritchard et al. 1997	0	0	1	0	1	1	0	0	0	0	1	0	0	1	1	1	0	0	1	6	40,00	8	42,11
Pritchard et al. 1996	0	0	1	0	1	1	0	0	0	0	1	0	0	1	1	1	0	0	0	6	40,00	7	36,84
Ross et al. 1996	1	1	0	1	1	0	1	0	0	0	1	0	0	1	1	1	0	0	1	8	53,33	10	52,63
Schwartz 1987	0	1	0	1	1	0	0	0	0	0	1	0	0	1	1	1	0	0	1	6	40,00	8	42,11
Schwartz et al. 1990	0	1	0	1	0	0	0	0	0	0	1	0	0	1	1	1	0	0	1	6	40,00	7	36,84
Stefanick et al. 1998	0	0	1	0	0	0	0	0	0	1	1	0	0	0	0	0	0	0	0	3	20,00	3	15,79
Svendsen et al. 1993	1	1	1	1	1	0	0	0	0	0	1	0	0	1	1	1	0	0	1	8	53,33	10	52,63
Svendsen et al. 1996	1	0	0	0	0	0	0	0	0	0	0	0	0	1	1	1	0	0	1	4	26,67	5	26,32
Thong et al. 2000	1	0	0	0	1	0	0	0	0	0	1	0	0	1	1	1	0	0	1	5	33,33	7	36,84
Wadden et al. 1997	1	1	0	1	0	0	0	1	0	1	1	0	1	1	1	1	0	0	1	10	66,67	11	57,89
Whatley et al. 1994	0	1	0	1	1	0	0	1	0	0	1	0	0	1	1	1	0	0	1	7	46,67	9	47,37
Wing et al. 1988 (1)	0	0	1	0	0	1	0	0	0	1	1	0	0	1	0	0	0	0	0	5	33,33	5	26,32
Wing et al. 1988 (2)	0	1	1	1	0	1	0	0	1	0	1	0	0	1	1	0	0	0	1	8	53,33	9	47,37
Wing et al. 1998	0	1	0	1	0	0	0	1	0	0	1	0	0	0	0	0	0	0	1	4	26,67	5	26,32
Wood et al. 1988	0	0	1	0	1	0	0	0	0	1	1	0	0	1	1	1	0	0	0	6	40,00	7	36,84
Wood et al. 1991	0	0	0	0	0	0	0	1	0	0	1	0	0	1	1	1	0	0	0	5	33,33	5	26,32
Described Total	25	15	9	26	22	5	8	21	3	6	36	1	11	44	43	42	3	0	22	6,32	42,13	7,2766	38,30
Described (%)	53,19	31,91	19,15	55,32	46,81	10,64	17,02	44,68	6,38	12,77	76,60	2,13	23,40	93,62	91,49	89,36	6,38	0,00	46,81				

Supplementary Table 4: CERT Results Reviewer 2.

Item / Study	1 WHAT (Material)	2 WHO: provider	3 HOW: delivery (individual or group)	4 HOW: delivery (supervision)	5 HOW: delivery (report and measure of adherence)	6 HOW: delivery (motivation strategies)	7a HOW: delivery (progression rules)	7b HOW: delivery (progression description)	8 HOW: delivery (exercise description)	9 HOW: delivery (home program)	10 HOW: delivery (non-exercise components)	11 HOW: delivery (adverse events)	12 WHERE: location	13 WHEN, HOW MUCH: dosage	14a TAILORING: what	14b TAILORING: how	15 TAILORING: starting level	16a HOW WELL: planned	16b HOW WELL: actual	Core Items (1-4, 6-10, 12-15) Total	Core Items (%)	All Items Total	All Items (%)
Ahmadizad et al. 2007	0	0	0	0	0	0	0	0	0	0	0	0	0	1	1	1	0	0	0	3	20,00	3	15,79
Ballor et al. 1996	0	0	1	1	0	0	0	1	0	0	1	0	0	1	1	1	1	0	0	8	53,33	8	42,11
Banz et al. 2003	1	0	1	1	1	1	0	1	0	1	1	0	1	1	1	1	0	0	1	11	73,33	13	68,42
Bateman et al. 2011	0	0	0	1	1	0	0	1	0	1	1	0	0	1	1	1	1	0	1	8	53,33	10	52,63
Davidson et al. 2009	0	0	0	1	0	0	0	1	0	0	1	0	0	1	1	1	0	0	1	6	40,00	7	36,84
Donges et al. 2010	0	1	0	1	1	0	0	1	0	0	1	0	1	1	1	1	1	0	1	9	60,00	11	57,89
Fenkci et al. 2006	1	0	0	0	0	0	0	1	0	0	0	0	0	1	1	1	0	0	0	5	33,33	5	26,32
Sarsan et al. 2006	1	0	0	1	0	0	0	1	0	0	1	0	1	1	1	1	1	0	0	9	60,00	9	47,37
Fisher et al. 2011	0	0	0	1	0	0	0	1	0	0	1	0	1	1	1	1	0	0	0	7	46,67	7	36,84
Janssen et al. 2002	0	1	0	1	0	1	0	1	0	1	0	0	0	1	1	1	1	0	1	9	60,00	10	52,63
Martins et al. 2010	1	1	0	1	0	0	0	1	0	0	0	0	0	0	0	0	0	0	0	4	26,67	4	21,05
Potteiger et al. 2012	0	1	1	1	0	1	0	1	0	0	1	0	1	0	1	1	0	0	1	9	60,00	10	52,63
Rice et al. 1999	0	0	0	1	0	1	0	1	0	0	1	0	0	1	1	1	0	0	1	7	46,67	8	42,11
Ross et al. 1994	0	0	0	1	0	1	0	1	0	0	1	0	0	1	1	1	0	0	1	7	46,67	8	42,11
Stensvold et al. 2010	0	1	0	1	0	0	0	0	0	0	1	1	1	1	1	1	1	0	1	8	53,33	10	52,63
Wallace et al. 1997	0	1	0	1	0	0	0	1	0	1	1	0	0	1	1	1	1	0	1	9	60,00	10	52,63
Willis et al. 2012	0	0	0	1	1	0	0	1	0	0	0	0	0	1	1	1	1	0	1	6	40,00	8	42,11
Aggel-Leijsen et al. 2001	1	1	0	1	1	0	0	0	0	1	1	0	1	1	1	1	1	0	1	10	66,67	12	63,16
Andersen et al. 1999	1	1	1	1	0	0	1	1	0	1	1	0	1	1	1	1	0	0	1	12	80,00	13	68,42
Anderssen et al. 1996	0	0	0	0	1	0	0	0	0	0	0	0	0	0	1	1	1	0	1	3	20,00	5	26,32
Cox et al. 2004	1	1	0	1	0	1	0	0	0	0	1	0	0	1	1	1	1	0	1	9	60,00	10	52,63
Gordon et al. 1997	0	1	0	0	1	1	0	0	0	1	1	1	1	1	1	1	1	0	0	9	60,00	11	57,89
Hays et al. 2004	1	0	0	1	0	0	0	0	1	0	1	0	0	1	1	1	1	0	0	8	53,33	8	42,11
Hellenius et al. 1993	1	1	0	1	1	0	0	0	1	1	1	0	0	1	1	1	1	0	1	10	66,67	12	63,16
Irwin et al. 2003	0	1	1	1	1	1	0	1	0	1	1	0	1	0	1	1	1	0	1	11	73,33	13	68,42
Jakicic et al. 1995	0	0	0	0	1	0	0	1	1	0	1	0	0	1	1	1	1	0	1	7	46,67	9	47,37
Jakicic et al. 2003	1	0	0	1	1	1	0	1	1	0	0	0	0	1	1	1	0	0	1	8	53,33	10	52,63
Kiernan et al. 2001	0	0	0	1	1	0	0	1	0	0	1	0	0	1	1	1	0	0	0	6	40,00	7	36,84
Leutholtz et al. 1995	0	0	0	0	1	1	0	0	1	0	1	0	0	1	1	1	1	0	1	7	46,67	9	47,37
Neumark-Sztainer et al. 1995	0	1	0	1	1	0	0	0	0	0	1	0	1	0	0	0	0	0	1	4	26,67	6	31,58
Nieman et al. 1998	1	0	0	1	1	0	0	1	0	1	1	0	1	0	1	1	0	0	1	8	53,33	10	52,63
Pritchard et al. 1997	0	0	0	1	1	1	0	0	1	1	1	0	0	1	1	1	1	0	1	9	60,00	11	57,89
Pritchard et al. 1996	0	0	0	0	1	1	0	0	1	0	1	0	0	1	1	1	1	0	1	7	46,67	9	47,37
Ross et al. 1996	0	1	0	1	0	1	0	1	0	0	0	0	0	0	1	1	0	0	1	6	40,00	7	36,84
Schwartz 1987	0	1	0	1	1	0	0	0	0	0	1	0	0	1	1	1	1	0	1	7	46,67	9	47,37
Schwartz et al. 1990	0	1	0	1	0	0	0	0	0	0	1	0	0	1	1	1	1	0	1	7	46,67	8	42,11
Stefanick et al. 1998	0	0	1	1	0	0	0	0	1	1	1	0	0	0	0	0	0	0	0	5	33,33	5	26,32
Svendsen et al. 1993	0	1	1	1	1	1	0	1	0	0	1	0	0	1	1	1	0	0	1	9	60,00	11	57,89
Svendsen et al. 1996	0	0	0	1	0	0	0	1	0	0	1	0	0	1	1	1	0	0	1	6	40,00	7	36,84
Thong et al. 2000	1	0	0	1	1	0	0	0	1	0	1	0	0	1	1	1	1	0	1	8	53,33	10	52,63
Wadden et al. 1997	0	1	1	1	0	0	0	1	0	1	1	0	1	0	1	1	0	0	1	9	60,00	10	52,63
Whatley et al. 1994	0	1	0	1	1	1	0	1	0	0	1	0	0	1	1	1	1	0	1	9	60,00	11	57,89
Wing et al. 1988 (1)	1	0	1	0	0	1	0	1	0	1	1	0	0	1	1	1	0	0	1	9	60,00	10	52,63
Wing et al. 1988 (2)	0	1	1	1	0	1	0	0	1	1	1	0	0	0	1	1	0	0	1	9	60,00	10	52,63
Wing et al. 1998	0	1	1	1	0	1	0	1	1	1	1	0	0	0	1	1	0	0	0	10	66,67	10	52,63
Wood et al. 1988	0	0	1	1	1	0	0	1	0	1	0	0	0	1	1	1	1	0	0	8	53,33	9	47,37
Wood et al. 1991	0	0	1	1	1	0	0	1	1	0	1	0	0	1	1	1	0	0	0	8	53,33	9	47,37
Described Total	13	21	13	39	23	18	1	30	12	17	38	2	13	36	44	44	24	0	34	7,72	51,49	8,97872	47,26
Described (%)	27,66	44,68	27,66	82,98	48,94	38,30	2,13	63,83	25,53	36,17	80,85	4,26	27,66	76,60	93,62	93,62	51,06	0,00	72,34				

Sports Medicine International Open | DOI 10.1055/a-2596-2049 | © 2025. The Author(s).
